# Diagnostic criteria for gestational hyperglycemia in women from a public maternity hospital in Brazil

**DOI:** 10.20945/2359-4292-2026-0054

**Published:** 2026-05-17

**Authors:** Thais Mantovani Bernardo, Ênio Luis Damaso, Patrícia Moreira Gomes, Elaine Christine Dantas Moisés

**Affiliations:** 1 Departamento de Ginecologia e Obstetrícia, Faculdade de Medicina de Ribeirão Preto, Universidade de São Paulo, Ribeirão Preto, SP, Brasil; 2 Departamento de Medicina, Faculdade de Medicina de Bauru, Universidade de São Paulo, Bauru, SP, Brasil; 3 Departamento de Clínica Médica, Divisão de Endocrinologia e Metabolismo, Faculdade de Medicina de Ribeirão Preto, Universidade de São Paulo, Ribeirão Preto, SP, Brasil

**Keywords:** Pregnancy, gestational diabetes mellitus, prevalence, diagnostic criteria

## Abstract

**Objective:**

This study aimed to determine the prevalence of hyperglycemia using different diagnostic criteria for gestational diabetes mellitus.

**Subjects and methods:**

This cross-sectional study evaluated parturients attending a hospital complex from January to December 2017. Data were obtained retrospectively from medical records. Fasting glucose and 75-g oral glucose tolerance test results were used to assess the prevalence of hyperglycemia during pregnancy. The diagnostic criteria evaluated were: 1998 World Health Organization (WHO98), 2003 American Diabetes Association (ADA03), 2015 National Institute for Health and Care Excellence (NICE15), and 2015 International Federation of Gynecology and Obstetrics (FIGO15). The overall kappa coefficient was used to analyze agreement among the four sets of diagnostic criteria. All analyses were performed using SAS 9.2, and a significance level of 5% was adopted for all tests.

**Results:**

Data from 2,262 women were analyzed. The prevalence of hyperglycemia differed depending on the diagnostic criteria: 10.3% (FIGO15), 7.34% (NICE15), 3.1% (ADA03), and 5.7% (WHO98). The overall kappa coefficient of agreement was 0.61. Individual analysis of each set of diagnostic criteria showed a kappa coefficient indicating moderate agreement, with FIGO15 used as the gold standard.

**Conclusion:**

In this population of pregnant women, the prevalence of hyperglycemia varied according to the diagnostic criteria used, with the prevalence of the disease being lower as the criteria became stricter. Lastly, the criteria showed moderate agreement when compared collectively and individually to FIGO15.

## INTRODUCTION

Gestational diabetes mellitus (GDM) is defined as any degree of glucose intolerance first recognized during pregnancy ^(1)^. It is a significant public health concern due to its association with maternal and fetal complications during pregnancy, as well as its potential long-term consequences. GDM increases the risk of preterm birth, fetal macrosomia, preeclampsia, and neonatal complications ^(1^,^2)^.

The first diagnostic criteria for GDM were proposed by O’Sullivan and Mahan in 1964 ^(3)^. In 1998, the World Health Organization (WHO98) recommended using diagnostic thresholds for non-pregnant individuals: fasting glucose ≥126 mg/dL or 2-hour glucose ≥140 mg/dL following a 75-gram oral glucose tolerance test (75-g OGTT) ^(4)^. Subsequently, the American Diabetes Association (ADA03) suggested fasting glucose ≥95 mg/dL, 1-hour ≥180 mg/dL, and 2-hour ≥155 mg/dL, requiring at least two abnormal results ^(5)^. The Latin American Diabetes Association (ALAD08) and the National Institute for Health and Care Excellence (NICE15) later recommended fasting glucose ≥100 mg/dL and 2-hour glucose ≥140 mg/dL ^(6^,^7)^.

A significant advancement occurred with the Hyperglycemia and Adverse Pregnancy Outcome study in 2008, which included 25,505 pregnant women undergoing 75-g OGTT between 24 and 32 weeks of gestation. The study demonstrated that increases in maternal glucose levels, even below diabetic thresholds, were independently associated with adverse perinatal outcomes ^(8)^. Based on these findings, the International Association of Diabetes and Pregnancy Study Groups (IADPSG) recommended universal screening with fasting glucose at the first prenatal visit and a 75-g OGTT between 24 and 28 weeks of gestation ^(9)^.

Currently, diagnostic criteria for GDM proposed by the American Diabetes Association ^(5)^, the World Health Organization ^(4)^, the International Federation of Gynecology and Obstetrics (FIGO15) ^(10)^, and the Endocrine Society Clinical Practice Guidelines ^(11)^ are based on the results of the Hyperglycemia and Adverse Pregnancy Outcome study, with values corresponding to those recommended by IADPSG: one or more values equal to or above 92–125 mg/dL (fasting), 180 mg/dL (1 hour), and 153–199 mg/dL (2 hours) after anhydrous 75-g glucose load ^(9)^. The diagnosis of overt diabetes in pregnancy includes fasting glucose (≥126 mg/dL), 2-hour glucose (≥200 mg/dL), random glucose (≥200 mg/dL), or glycated hemoglobin (≥6.5%) ^(9)^. In Brazil, the Brazilian Federation of Gynecology and Obstetrics Associations, the Brazilian Diabetes Society, the Pan American Health Organization, and the Brazilian Ministry of Health have adopted the same IADPSG diagnostic recommendations in 2017 ^(12)^.

It is estimated that approximately 16% of live births occur in women who experienced hyperglycemia during pregnancy, with about 8% of these cases involving pre-existing diabetes ^(13)^. Therefore, given the limited data and ongoing debate regarding optimal diagnostic thresholds, this study aimed to determine the prevalence of hyperglycemia using different diagnostic criteria and to assess the agreement among them in a population of pregnant women.

## SUBJECTS AND METHODS

### Study design and period

This observational, cross-sectional study analyzed data from the hospital complex of Ribeirão Preto (São Paulo State, Brazil). The complex, managed by the University of São Paulo, comprises two units: the medium-complexity Referral Center for Women’s Health of Ribeirão Preto (CRSMRP-MATER) and the high-complexity University Hospital of the Ribeirão Preto Medical School (HCFMRP-USP). Both provide pregnancy and postpartum care through the Brazilian public health system, serving a population of approximately 1.4 million people. Ribeirão Preto is located in northeastern São Paulo State, having a human development index of 0.800 in 2010 and ranking 40th in Brazil ^(14)^. This study analyzed data from pregnant women attended between January and December 2017, coinciding with the adoption of Brazil’s updated diagnostic criteria. It was conducted as outlined by STROBE guidelines for reporting observational studies and adhered to the Declaration of Helsinki. Additionally, it was approved by the Research Ethics Committee of HCFMRP-USP and CRSMRP-MATER (CAAE no. 62213416.9.0000.5440).

### Participants

The study included pregnant women residing in Ribeirão Preto who delivered at CRSMRP-MATER or HCFMRP-USP in 2017. Only those who completed prenatal care and gave birth at these institutions were included, ensuring adherence to national diagnostic guidelines. Exclusion criteria included multiple pregnancies, previous diagnosis of type 1 or type 2 diabetes mellitus, and absence of prenatal care in Ribeirão Preto. For GDM prevalence analyses, women without fasting glucose or 75-g OGTT results were excluded. For the analysis of adverse outcomes, women with missing data on delivery, maternal, or perinatal outcomes were also excluded.

### Data collection

Data were collected retrospectively from medical records. Clinical and sociodemographic variables were retrieved, including maternal age, self-reported skin color, comorbidities, lifestyle habits, parity, previous cesarean sections, prenatal care, gestational age at delivery, and type of delivery. Fasting blood glucose in early pregnancy (up to 20 weeks) and the 75-g OGTT performed between 24 and 28 weeks were analyzed to assess the prevalence of hyperglycemia and diagnostic agreement. Four diagnostic criteria were applied: WHO98 ^(4)^, ADA03 ^(5)^, NICE15 ^(7)^, and FIGO15 ^(10)^. According to WHO98 criteria ^(4)^, gestational diabetes is diagnosed with fasting plasma glucose ≥126 mg/dL or 2-hour plasma glucose ≥140 mg/dL after a 75-g OGTT. ADA03 criteria ^(5)^ define GDM as two or more values meeting or exceeding the following thresholds during a 75-g OGTT: fasting ≥95 mg/dL, 1-hour ≥180 mg/dL, and 2-hour ≥155 mg/dL. NICE15 criteria ^(7)^ recommend diagnosing GDM when fasting plasma glucose is ≥100 mg/dL or 2-hour plasma glucose is ≥140 mg/dL after 75-g OGTT. The FIGO15 criteria ^(10)^, based on IADPSG recommendations ^(9)^, establish GDM diagnosis via a fasting plasma glucose ≥92 mg/dL, or one or more of the following OGTT thresholds: ≥92 mg/dL (fasting), ≥180 mg/dL (1 hour), or ≥153 mg/dL (2 hour). For clarity, the FIGO15 diagnostic criteria applied are fully equivalent to the IADPSG recommendations and have been the official diagnostic standard in Brazil since 2017 ^(9^,^10^,^12)^.

### Statistical analysis

Exploratory analyses were conducted to characterize the sample. Quantitative variables are summarized using measures of central tendency and dispersion, while qualitative variables are presented as absolute and relative frequencies. Agreement among the four diagnostic criteria was assessed using the kappa coefficient, as described by Cohen ^(15)^. A kappa value of 1 indicates perfect agreement, 0 indicates chance agreement, and negative values represent agreement below chance. Interpretation of kappa values followed the Landis and Koch ^(16)^ classification: poor (<0), negligible (0.00–0.20), weak (0.21–0.40), moderate (0.41–0.60), strong (0.61–0.80), and almost perfect (0.81–1.00). All statistical analyses were performed using SAS version 9.2 (SAS Institute, USA) using a significance level of 5%.

## RESULTS

**Figure 1.**
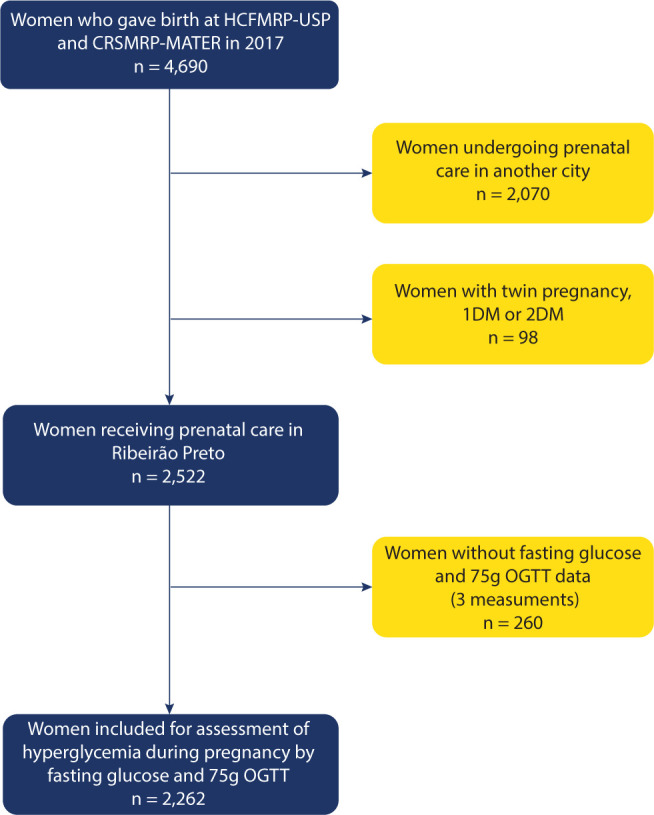
Flowchart depicting the study design.

From an initial 4,690 records, the final sample of 2,262 pregnant women was selected based on the availability of fasting plasma glucose and/or 75-g OGTT results. The sample selection process is summarized in **Figure 1**.

**Table 1** presents the clinical and sociodemographic characteristics of the study population. The mean maternal age was 26.4 years (SD ±5.7), with 70.2% aged 20–34 years. Regarding self-reported race, 65.2% identified as white and 34.3% as black. Educational attainment was high, with 65% having completed at least secondary education. Most women (93.9%) initiated prenatal care before 20 weeks, and 93.4% attended at least six prenatal consultations. The mean pregestational body mass index was 26.2 kg/m^2^, with 41.5% classified as being overweight or obese.

**Table 1.** Clinical and sociodemographic characteristics of women assessed for the prevalence of gestational hyperglycemia (n = 2,262)

Variable	n	%	Variable	n	%
Maternal age (years)			Prenatal (≥ 6 visits)		
Mean (SD)	26.41	(6.60)	No	83	3.67
Median (min–max)	26	(13-47)	Yes	2,112	93.37
Maternal age (years)			Not reported	67	2.96
< 20	372	16.44	Total number of pregnancies		
20–34	1589	70.25	1	777	34.35
> 34	301	13.31	2	642	28.38
Skin color			3	420	18.57
White	1,474	65.16	4	237	10.48
Black	776	34.31	≥ 5	86	8.22
Brown	7	0.31	Parity		
Yellow	2	0.09	0	885	39.12
Not reported	3	0.13	1	703	31.08
Drug use			2	393	17.37
Yes	254	11.23	3	175	7.74
No	2,008	88.77	4	58	2.56
Maternal diseases			≥ 5	48	2.12
None	1,371	60.6	Previous cesarean section		
Hypertensive disorders of pregnancy	244	10.78	0	1,742	77.01
			1	370	16.36
HIV	14	0.62	2	115	5.08
Others	632	27.93	3	32	1.41
Not reported	1	4	≥ 4	3	0.13

To evaluate potential selection bias, we compared the baseline characteristics of women who underwent screening with those who did not. Non-screened women were more likely to be older than 34 years, report higher rates of drug use, and have fewer than six prenatal visits. These differences suggest that the excluded population may have had a higher underlying risk for GDM. The detailed analysis is presented in **Supplementary Table 1**.

The prevalence of hyperglycemia during pregnancy was calculated for the full cohort (n = 2,262) using fasting glucose and/or OGTT data according to FIGO15, NICE15, WHO98, and ADA03 criteria. Of these, 1,772 women had complete three-point OGTT results, allowing for the application of all four criteria. For the remaining 490 women with only fasting glucose data, only the FIGO15, NICE15, and WHO98 criteria could be applied, as ADA03 requires complete OGTT data. **Table 2** lists the prevalence rates obtained with each diagnostic criterion. Among women with any available glucose data, the prevalence ranged from 3.1% (ADA03) to 10.3% (FIGO15). NICE15 and WHO98 criteria yielded intermediate rates of 7.3% and 5.7%, respectively. In the subgroup with complete OGTT data, prevalence was highest with FIGO15 (11.6%), followed by NICE15 (8.8%), WHO98 (6.3%), and ADA03 (3.9%).

**Table 2.** Prevalence of gestational hyperglycemia in women with fasting glucose and 75-g OGTT values according to each set of diagnostic criteria

Criteria	Fasting glucose and all 75g OGTT values		Individual fasting glucose and fasting glucose value extracted from 75-g OGTT		Three 75g OGTT values	
	n (%)	95% CI (%)	n (%)	95% CI (%)	n (%)	95% CI (%)
FIGO15						
No	2,029 (89.70)	88.45; 90.95	2,082 (92.04)	90.93; 93.16	1,615 (91.14)	89.82; 92.46
Yes	233 (10.30)	9.05; 11.55	180 (7.96)	6.84; 9.07	157 (8.86)	7.54; 10.18
NICE15						
No	2,096 (92.66)	91.59; 93.74	2,184 (96.55)	95.8; 97.3	1,641 (92.61)	91.39; 93.83
Yes	166 (7.34)	6.26; 8.41	78 (3.45)	2.7; 4.2	131 (7.39)	6.17; 8.61
ADA03						
No	2,191 (96.90)	96.19; 97.62	-	-	1,711 (96.56)	95.71; 97.41
Yes	71 (3.10)	2.38; 3.81	-	-	61 (3.44)	2.59; 4.29
WHO98						
No	2,133 (94.30)	93.34; 95.25	2,245 (99.25)	98.89; 99.6	1,657 (93.51)	92.36; 94.66
Yes	129 (5.70)	4.75; 6.66	17 (0.75)	0.4; 1.11	115 (6.49)	5.34; 7.64
GDM diagnosis by some of the criteria						
No	1,998 (88.33)	87.01; 89.65	2,082 (92.04)	90.93; 93.16	1,586 (89.50)	88.08; 90.93
Yes	264 (11.67)	10.35; 12.99	180 (7.96)	6.84; 9.07	186 (10.50)	9.07; 11.92

**Figure 2.**
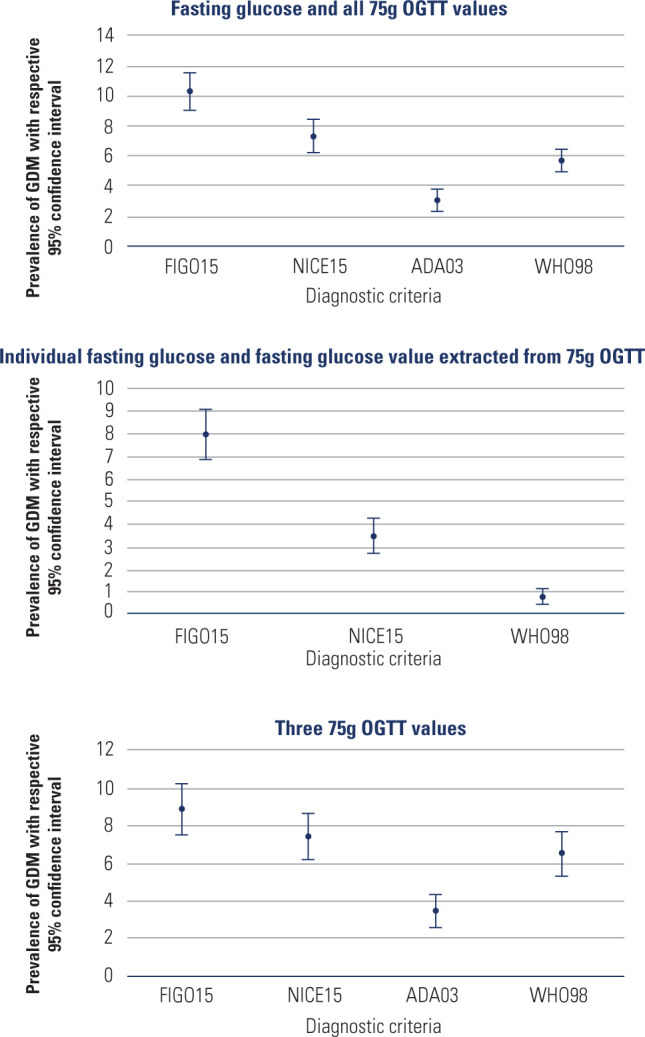
Prevalence of gestational hyperglycemia in women with fasting glucose and 75-g OGTT values according to each set of diagnostic criteria

The prevalence of these variations across diagnostic criteria are illustrated in **Figure 2**.

**Table 3** presents the absolute margin of error for prevalence estimates, considering both the total sample and the subgroup with complete OGTTs. The margins ranged from 0.71 to 1.32 percentage points, depending on criterion and sample size. Agreement between diagnostic sets was evaluated using kappa statistics. The overall kappa among the four criteria was 0.61, indicating moderate agreement. When compared individually to FIGO15 as the reference, kappa values were 0.65 for NICE15, 0.51 for WHO98, and 0.44 for ADA03.

**Table 3.** Estimation of the margin of error based on the sample

Variable	Diagnostic criteria	Prevalence of hyperglycemia	n	Absolute error (pp)
Fasting glucose and all 75-g OGTT values	FIGO15	233 (10.3%)	2,262	1.25
	NICE15	166 (7.34%)	2,262	1.07
	ADA03	70 (3.1%)	2,261	0.71
	WHO98	129 (5.7%)	2,262	0.96
	S-GDM	264 (11.67%)	2,262	1.32
Individual fasting glucose and fasting glucose value extracted from 75-g OGTT	FIGO15	180 (7.96%)	2,262	1.12
	NICE15	78 (3.45%)	2,262	0.75
	WHO98	17 (0.75%)	2,262	0.36
	S-GDM	180 (7.96%)	2,262	1.12
Three 75-g OGTT values	FIGO15	157 (8.86%)	1,772	1.32
	NICE15	131 (7.39%)	1,772	1.22
	ADA03	61 (3.44%)	1,772	0.85
	WHO98	115 (6.49%)	1,772	1.15
	S-GDM	186 (10.5%)	1,772	1.43

## DISCUSSION

This study evaluated the medical records of women who received prenatal care through the Brazilian public health system and gave birth at HCFMRP-USP and CRSM-MATER. In 2017, the FIGO15 criteria were already recommended in Ribeirão Preto.

Prevalence of hyperglycemia varied across diagnostic criteria: 10.3% (FIGO15), 7.3% (NICE15), 3.1% (ADA03), and 5.7% (WHO98). This highlights the direct impact of the diagnostic strategy on the proportion of women identified with GDM and its implications for clinical practice, public health policy, and international comparability of data. Since the initial recognition of hyperglycemia in pregnancy, the absence of a universal diagnostic standard has hindered accurate estimates of GDM prevalence ^(9)^. Broad early definitions, encompassing any degree of hyperglycemia first recognized during pregnancy, contributed to discrepancies across studies.

Globally, GDM prevalence estimates vary widely from 1% to 37.7%, with a mean prevalence of 16.2% ^(10^,^17)^. The International Diabetes Federation reports a pooled global standardized prevalence of 14.0%. By region, standardized GDM prevalence is 7.1% in North America and the Caribbean, 7.8% in Europe, 10.4% in South and Central America, 14.2% in Africa, 14.7% in the Western Pacific, 20.8% in South-East Asia, and 27.6% in the Middle East and North Africa. According to economic status, the standardized prevalence is 12.7% in low-income countries, 9.2% in middle-income countries, and 14.2% in high-income countries ^(18)^. A meta-analysis indicated an overall pooled prevalence of 4.4%, while specifically applying FIGO15 criteria resulted in 10.6% ^(19)^, consistent with our findings.

An investigation comparing various diagnostic criteria found that GDM prevalence ranged from 9.2% to 45.3%, depending on the thresholds used ^(20)^. Adoption of the IADPSG criteria significantly increased GDM detection, and prevalence rates can vary by a factor of 1.5 to 4.9. In Brazil, data are limited and divergent. A reanalysis of a cohort from 1991 to 1995 showed a GDM prevalence of 18% when using IADPSG criteria, which decreased to 2.7% under stricter definitions ^(21)^. A 2024 systematic review reported a pooled prevalence of 14% (95% CI: 11.0–16.0) for GDM in Brazil ^(22)^. Although more sensitive criteria (i.e., IADPSG) identify additional cases, this increase does not necessarily result in more adverse outcomes.

Among the evaluated criteria, FIGO15 consistently showed the highest prevalence, as expected due to its broader diagnostic thresholds. Conversely, ADA03 was the most restrictive, requiring two abnormal glucose values and thus producing lower prevalence rates, aligning with findings from other studies, such as that by Çelik and cols. ^(23)^, which confirmed that broader thresholds akin to those of FIGO15 significantly increase detection rates. Although IADPSG and FIGO15 criteria increase GDM diagnosis rates, they improve maternal and neonatal outcomes and reduce healthcare costs. A Spanish study demonstrated that use of IADPSG criteria led to increased detection, improved pregnancy outcomes, and cost savings ^(24)^.

Agreement analyses showed moderate concordance among diagnostic sets, supporting the systematic differences found. Notably, agreement between FIGO15 and WHO98 was low when only fasting glucose was available. The FIGO15 criteria are notable for being the first to base diagnosis on perinatal outcomes, facilitating early intervention and influencing maternal and neonatal morbidity and mortality. Early diagnosis of overt diabetes also enables more intensive management; for example, maintaining glycated hemoglobin below 6% reduces risks of large-for-gestational-age infants, preterm delivery, and preeclampsia ^(17)^.

Despite our promising findings, this study’s limitations include its retrospective, observational design, possible selection bias, and potential confounding factors. The use of a convenience sample also limited the generalizability of findings, highlighting the need for multicenter studies to better characterize GDM prevalence across Brazil’s diverse population.

In conclusion, the prevalence of gestational hyperglycemia varied according on the diagnostic criteria, with ADA03 yielding the lowest and FIGO15 the highest prevalence. Broader criteria such as FIGO15 enhance early identification and allow for timely intervention, potentially improving maternal and neonatal outcomes. These findings support public health priorities in low- and middle-income countries.

## Data Availability

data related to this article will be available upon request to the corresponding author.

## SUPPLEMENTARY MATERIAL

**Supplementary Table 1.** Clinical and sociodemographic characteristics of screened vs. non-screened women who gave birth at HCFMRP-USP and CRSM-MATER in 2017

Variable	Not screened (n = 260)	Screened (n = 2,262)	p*
Maternal age (years)			
< 20	25 (9.62%)	314 (13.88%)	<0.01
20–34	166 (63.85%)	1,600 (70.73%)	
> 34	69 (26.54%)	348 (15.38%)	
Skin color^‡^			
White	191 (73.46%)	1,474 (65.25%)	0.05
Black	69 (26.54%)	776 (34.35%)	
Brown	0 (0%)	7 (0.31%)	
Yellow	0 (0%)	2 (0.09%)	
Drug use			
No	44 (16.92%)	2,008 (88.77%)	<0.01
Yes	216 (83.08%)	254 (11.23%)	
Maternal diseases			
None	157 (60.38%)	1,371 (60.64%)	0.45
Hypertensive disorders of pregnancy	24 (9.23%)	244 (10.79%)	
HIV	0 (0%)	14 (0.62%)	
Others	79 (30.38%)	632 (27.95%)	
Prenatal (≥ 6 visits)			
No	37 (14.23%)	83 (3.78%)	<0.01
Yes	223 (85.77%)	2,112 (96.22%)	
Total number of pregnancies			
1	85 (32.69%)	777 (34.35%)	0.04
2	75 (28.85%)	642 (28.38%)	
3	35 (13.46%)	420 (18.57%)	
4	32 (12.31%)	237 (10.48%)	
≥ 5	33 (12.69%)	186 (8.22%)	
Parity			
0	107 (41.15%)	885 (39.12%)	0.01
1	74 (28.46%)	703 (31.08%)	
2	34 (13.08%)	393 (17.37%)	
3	20 (7.69%)	175 (7.74%)	
4	14 (5.38%)	58 (2.56%)	
≥ 5	11 (4.23%)	48 (2.12%)	
Previous cesarean section			
0	200 (76.92%)	1,742 (77.01%)	<0.01
1	36 (13.85%)	370 (16.36%)	
2	14 (5.38%)	115 (5.08%)	
3	6 (2.31%)	32 (1.41%)	
≥ 4	4 (1.54%)	3 (0.13%)	

* Chi-square test;

‡ Fisher’s exact test.

## References

[1] Sweeting A, Wong J, Murphy HR, Ross GP (2022). A Clinical Update on Gestational Diabetes
Mellitus. Endocr Rev..

[2] Li T, Ma X, Ma L (2025). Effects of gestational diabetes mellitus on offspring: A
literature review. Int J Gynaecol Obstet..

[3] O’Sullivan JB, Mahan CM (1964). Criteria for the oral glucose tolerance test in
pregnancy. Diabetes.

[4] Alberti KG, Zimmet PZ (1998). Definition, diagnosis and classification of diabetes mellitus and
its complications. Part 1: diagnosis and classification of diabetes mellitus
provisional report of a WHO consultation. Diabet Med..

[5] American Diabetes Association (2003). Gestational diabetes mellitus. Diabetes Care.

[6] Asociación Latinoamericana de Diabetes (ALAD) (2008). Consenso Latinoamericano de Diabetes y Embarazo. Rev ALAD.

[7] National Collaborating Centre for Women’s and Children’s Health
(UK) (2015). Diabetes in pregnancy: management of diabetes and its complications from
preconception to the postnatal period.

[8] Metzger BE, Lowe LP, Dyer AR, Trimble ER, Chaovarindr U, Coustan DR, HAPO Study Cooperative Research Group (2008). Hyperglycemia and adverse pregnancy outcomes. N Engl J Med..

[9] Metzger BE, Gabbe SG, Persson B, Buchanan TA, Catalano PA, Damm P, International Association of Diabetes and Pregnancy Study Groups
Consensus Panel (2010). International association of diabetes and pregnancy study groups
recommendations on the diagnosis and classification of hyperglycemia in
pregnancy. Diabetes Care.

[10] Hod M, Kapur A, Sacks DA, Hadar E, Agarwal M, Di Renzo GC (2015). The International Federation of Gynecology and Obstetrics (FIGO)
Initiative on gestational diabetes mellitus: A pragmatic guide for
diagnosis, management, and care. Int J Gynaecol Obstet..

[11] Blumer I, Hadar E, Hadden DR, Jovanović L, Mestman JH, Murad MH, Yogev Y (2013). Diabetes and pregnancy: an endocrine society clinical practice
guideline. J Clin Endocrinol Metab..

[12] Zajdenverg L, Façanha C, Dualib P, Golbert A, Moisés E, Calderon I (2023). Rastreamento e diagnóstico da hiperglicemia na
gestação: Diretriz oficial da Sociedade Brasileira de Diabetes
– 2023.

[13] International Diabetes Federation (2019). IDF Diabetes Atlas.

[14] Instituto Brasileiro de Geografia e Estatística
(IBGE) (2020). Estimativa populacional 2020.

[15] Cohen J (1960). A coefficient of agreement for nominal scales. Educ Psychol Meas..

[16] Landis JR, Koch GG (1977). The measurement of observer agreement for categorical
data. Biometrics.

[17] McIntyre HD, Catalano P, Zhang C, Desoye G, Mathiesen ER, Damm P (2019). Gestational diabetes mellitus. Nat Rev Dis Primers.

[18] Wang H, Li N, Chivese T, Werfalli M, Sun H, Yuen L (2022). IDF Diabetes Atlas: estimation of global and regional gestational
diabetes mellitus prevalence for 2021 by International Association of
Diabetes in Pregnancy Study Groups criteria. Diabetes Res Clin Pract..

[19] Behboudi-Gandevani S, Amiri M, Bidhendi Yarandi R, Ramezani Tehrani F (2019). The impact of diagnostic criteria for gestational diabetes on its
prevalence: a systematic review and meta-analysis. Diabetol Metab Syndr..

[20] Agarwal MM, Dhatt GS, Othman Y (2015). Gestational diabetes: differences between the current
international diagnostic criteria and implications of switching to
IADPSG. J Diabetes Complications.

[21] Trujillo J, Vigo A, Duncan BB, Falavigna M, Wendland EM, Campos MA (2015). Impact of the International Association of Diabetes and Pregnancy
Study Groups criteria for gestational diabetes. Diabetes Res Clin Pract..

[22] Mocellin LP, Gomes HA, Sona L, Giacomini GM, Pizzuti EP, Nunes GB (2024). Gestational diabetes mellitus prevalence in Brazil: a systematic
review and meta-analysis. Cad Saude Publica..

[23] Çelik C, Oral E, Özdoğan E, Atak M, Kumbak B, Karslı MF (2021). Comparison of different diagnostic criteria for gestational
diabetes mellitus in Turkish pregnant women: prevalence, maternal and
neonatal outcomes. J Obstet Gynaecol Res..

[24] Duran A, Sáenz S, Torrejón MJ, Bordiú E, Del Valle L, Galindo M (2014). Introduction of IADPSG criteria for the screening and diagnosis
of gestational diabetes mellitus results in improved pregnancy outcomes at a
lower cost in a large cohort of pregnant women: the St. Carlos Gestational
Diabetes Study. Diabetes Care.

